# In Memoriam: Ahmed Hassan Zewail (1946–2016)

**DOI:** 10.1063/1.4961968

**Published:** 2016-08-31

**Authors:** Majed Chergui

**Affiliations:** Laboratory of Ultrafast Spectroscopy (LSU), Lausanne Centre for Ultrafast Science (LACUS), Ecole Polytechnique Fédérale de Lausanne, ISIC, FSB, Station 6, CH-1015 Lausanne, Switzerland

It is with great sorrow that we heard of the passing away of Ahmed H. Zewail, the father of Femtochemistry and the pioneer of ultrafast electron-based Science, on August 2, 2016. This represents a tremendous loss not only for the scientific community but also for humanity, as he was a real Statesman.

In 1987, Ahmed Zewail published a short communication in the Journal of Chemical Physics^1^ that sparked off a revolution in Science. Shortly afterwards, two articles were published in the same journal,^2,3^ presenting a more complete account, both technical and scientific, of the “real-time” probing of a chemical reaction, in this case, the photodissociation of the ICN molecule, with femtosecond resolution. This was the beginning of breathtaking series of discoveries, which represented the birth of a new branch of Chemistry, Femtochemistry, for which he was awarded the Nobel Prize for Chemistry in 1999.

Ahmed Zewail was born in 1946 in Damanhur, Delta of the Nile, Egypt. He obtained his Master's degree from the University of Alexandria in 1969. Inspired by the style of one of his mentors, he decided to move to the United States for his Ph.D. studies. He did his thesis under the supervision of the late Robin Hochstrasser at the University of Pennsylvania and obtained the doctoral degree in 1973. Thereafter, Zewail became a postdoctoral research assistant for two years at the University of California, Berkeley, in the group of Charles B. Harris. During that time he started to work with the newly developed picosecond lasers, the springboard to the new research that he initiated at Caltech in 1976 as an assistant professor.

In Caltech, he developed his concepts about coherence, and in a series of papers—which earned him tenure after two years—he pioneered studies of coherence and dephasing with lasers in the case of both isolated or the condensed phase molecules. This work served as a seed to the birth of Femtochemistry. Indeed, even on the short picosecond time scale, molecular states already reside in eigenstates (the static limit) and there is only one evolution possibly observable: the change of population with the time of that state. Hence, with picosecond resolution, one was still concerned with kinetics, not dynamics. The advent of the femtosecond laser technology in the mid-1980s, thanks to the works of C. V. Shank and his co-workers, opened the door to the above mentioned breakthrough in 1987–1988 in which he demonstrated the “real-time” visualization of nuclear motion in molecular systems. Ahmed Zewail's genius was his ability to convey the power and insight of the new discoveries by a judicious choice of the systems to study, going from simple ones to systems of ever greater complexity. With this powerful tool in hand, Zewail and co-workers could observe the dynamical processes of bond-breaking, bond making, and of the cornerstone of reactivity, the transition state, with atomic scale resolution. S. Forsen, a Swedish researcher of Lund University, likened the situation, before the advent of Femtochemistry, to an audience seeing only the very beginning and the very end of Hamlet “the main characters are introduced, then the curtain falls for a change of scenery, and, as it rises again, we see on the stage a considerable number of bodies and a few survivors. Not an easy task for the unexperienced to unravel what actually took place.” But with Ahmed Zewail's achievements, the stage was set to see the whole action!

The impact of his discoveries went quickly beyond chemistry as they dealt with the “atomic-scale of time” (the tens to hundreds of femtoseconds), opening new perspectives for research in Biology, Condensed Matter Physics, and Materials Science. This, along with his enthusiasm and his sense of communication, brought about a real revolution in science, which was recognised by the Nobel Committee in 1999, barely 12 years after Ahmed's first pioneering experiments.

During his Nobel lecture, Ahmed made a humoristic remark about the Nobel Prize citation for not mentioning his work on ultrafast electron diffraction and scattering, Indeed, early in 1990, when he was starting the “Femtochemistry revolution,” was he aware of the fact that optical-domain (ultraviolet, visible, and infrared) spectroscopy does not deliver structures, except in a very few rare cases such as diatomic molecules. Therefore, he combined the atomic-scale temporal resolution of femtosecond lasers with the atomic-scale spatial resolution of electron-based structural methods (diffraction and microscopy). This was a daring and visionary choice, which now fully recovers its importance and depth. Indeed, electrons have orders of magnitude larger scattering cross-sections than X-rays, as well as a much reduced energy deposition per scattering event. Finally, they are much easier to manipulate by electron optics than X-rays are. While most of the community of ultrafast scientists (including the author of this piece) adopted ultrashort pulses of X-rays, he was the only scientist to go for ultrashort pulses of electrons. This implied overcoming a number of serious technical challenges, which were never an issue for him. Just as with the discoveries leading to Femtochemistry, he adopted a systematic strategy starting with simple molecular systems in the gas phase, then increasing the complexity of the molecular systems under study. By the early 2000, he achieved a great leap forward: the study by ultrafast electron diffraction and crystallography of dynamical processes in condensed phases, such as interfacial water on a hydrophilic surface, adsorbates on surfaces, and important to Biology, phospholipids and fatty acid bilayers.

Almost at the same time, he achieved yet another great leap forward with the pioneering of real-time and real-space electron microscopy or four-dimensional electron microscopy (4D-EM). This represents a real revolution in the field of structural dynamics. This technique is delivering an amazing degree of insight into the mechanisms behind a large class of phenomena in biology, condensed matter physics, materials science, and nanoscience.

What's most impressive with Ahmed Zewail's achievements using electron-based techniques is the number of new avenues he has opened with each of his breakthroughs. New methods have emerged from his labs, such as ultrafast Electron Energy Loss Spectroscopy (EELS). Along with 4D-EM, these two tools deliver the full dynamical and electronic information in real-time, real-space, and in energy-space. He also introduced the so-called Photon-induced near-field electron microscopy (PINEM), which allows the visualization of the evanescent electric fields around the excited species. PINEM has turned into a major tool for visualizing the time evolution of nano-sized objects in Biology and Materials Science, and it is now making its way into the scientific community worldwide. Parallel to this, he also demonstrated the impressive results of electron nano-diffraction, opening the way to the study of single nano-objects and of powders. Despite his illness, which appeared in 2013, Ahmed Zewail went on widening the range and scope of systems one can study with 4D-EM, diffraction, and related methods in Biology, Nanoscience, and Materials Science. There is no doubt in the opinion of the author (and of many colleagues) that he was on his way to a second Nobel Prize for his outstanding achievements using electron-based techniques. Ahmed Zewail had enthusiastically accepted to become a member of the Advisory Board of Structural Dynamics, when the journal was launched in 2014. He already published two articles in the journal,^4,5^ which undoubtedly represent breakthroughs in the field of electron-based Science.^6^

His innumerable scientific achievements (about 600 articles and over a dozen books) earned him an impressive number (more than 100) of prizes, awards, and honorary degrees that are impossible to list here. His former students and postdoctoral researchers (his “Science Family” as he liked to put it) can be found in leading positions in Science around the Globe.

Ahmed Zewail's scientific achievements cannot be disconnected from his personality, and his concerns for others. Despite reaching the summits of Fame and Glory, he was always available, attentive, and respectful of people's opinion and points of view. I remember calling him several times the day following the news of his earning the Nobel Prize, and when I finally got his secretary on the line, she told me he was discussing a paper with two of his postdocs! The following day, I got a call from him in the morning (it must have been 1 AM in California) to thank me about my call! I was really amazed, why would he bother when he had the entire world over him?

Ahmed Zewail took to heart the conditions and needs of the “have-nots,” as he used to say. His concerns were directed especially towards the Arab World and more specifically towards his country of origin, Egypt. He considered the support for Science in the developing world of paramount importance for the betterment of its humanity (part of his Nobel Prize was donated to provide scholarships in Egypt). He had been actively working to this aim, already prior to him getting the Nobel Prize but then more actively after. In Egypt (and in the broader Arab world), his popularity was much higher than that of cinema, music or sports stars (not to say of politicians…), and whenever we met, I witnessed how often he would be greeted by people congratulating him in Arabic for the Nobel Prize but also for giving them Pride. And he always took the time to return his thanks and gratitude. He became an ambassador of Science in the world even prior to being appointed United States First Science Envoy to the Middle East (2009–2011), President Obama's Council of Advisors on Science and Technology (2009–2013), and UN Secretary General Ban Ki-moon's Scientific Advisory Board (2013-). During the recent events in Egypt, he made it clear that he had no political ambitions. Indeed, it was a leitmotiv of his that the best way to serve Egypt and the Arab world was to pursue excellence in Science. As a matter of fact, his long-term goal of building a world class Science and Technology University in Egypt, the Zewail City, was reached after years of hard work through the meanders of bureaucratic and political difficulties, especially in the recent years.

Ahmed Zewail touched the lives of countless people in and out of the scientific arena and he leaves an immense legacy, be it scientific, societal and humanistic. His passing away creates a great void and we miss him greatly, but his influence is deep, broad, and it will last for generations. In this respect, he is of this world for ever.
